# Effect of Cyclohexane on the Combustion Characteristics of Multi-Component Gasoline Surrogate Fuels

**DOI:** 10.3390/molecules28114273

**Published:** 2023-05-23

**Authors:** Shunlu Rao, Zhaolei Zheng, Chao Yang

**Affiliations:** Key Laboratory of Low-Grade Energy Utilization Technologies and Systems, Ministry of Education, School of Energy and Power Engineering, Chongqing University, Chongqing 400044, China; raoshunlu@cqu.edu.cn (S.R.); mryangchao@cqu.edu.cn (C.Y.)

**Keywords:** gasoline surrogate, cyclohexane, ignition-delay time, laminar flame speed

## Abstract

It has been discovered that there is a dynamic coupling between cycloalkanes and aromatics, which affects the number and types of radicals, thereby controlling the ignition and combustion of fuels. Therefore, it is necessary to analyze the effects of cyclohexane production in multicomponent gasoline surrogate fuels containing cyclohexane. In this study, a five-component gasoline surrogate fuel kinetic model containing cyclohexane was first verified. Then, the effect of cyclohexane addition on the ignition and combustion performance of the surrogate fuel was analyzed. This study shows that the five-component model exhibits good predictive performance for some real gasoline. Meanwhile, the addition of cyclohexane decreases the ignition-delay time of the fuel in the low and high temperature bands, which is caused by the early oxidation and decomposition of cyclohexane molecules, generating more OH radicals; in the medium temperature band, the isomerization and decomposition reactions of cyclohexane oxide cC_6_H_12_O_2_ dominate the temperature sensitivity of the ignition delay, affecting the small molecule reactions that promote the generation of reactive radicals such as OH, thus inhibiting the negative temperature coefficient behavior of the surrogate fuel. The laminar flame speed of the surrogate fuels increased with the increase in the proportion of cyclohexane. This is due to the fact that the laminar flame speed of cyclohexane is higher than that of chain and aromatic hydrocarbons, and the addition of cyclohexane dilutes the ratio of chain and aromatic hydrocarbons in the mixture. In addition, engine simulation studies have shown that at higher engine speeds, the five-component surrogate fuel containing cyclohexane requires lower intake-gas temperatures to achieve positive ignition and are closer to the in-cylinder ignition of real gasoline.

## 1. Introduction

With the growing world population and economy, the demand for energy is also increasing. The transportation sector is one of the major final consumers of total energy and a contributor to global atmospheric emissions. This sector accounts for 29% of the total world energy consumption and 65% of the world’s consumption of petroleum products, while gasoline is one of the most consumed fuels in the transportation sector [1,2]. In addition, some studies have pointed out that the structure of energy consumption will not undergo major changes for a long period of time in the future. Although the share of renewable energy is increasing, oil, natural gas, and coal will still dominate [3]. Energy conservation and environmental protection are two important themes in today’s world economic development. The efficient utilization of fossil energy can not only reduce energy waste but also reduce greenhouse gas emissions and then alleviate global warming. Therefore, it is still of great significance to study the efficient utilization of fossil fuels.

Gasoline, which is mainly composed of alkanes, naphthenes, olefins, and aromatic hydrocarbons, is a mixture of hydrocarbons with very complex components [4]. Gasoline components vary from country to country, from crude oil source to crude oil source, and from refining process to refining process. Gasoline fuels with different components have different physical properties (such as density and viscosity) and chemical properties (such as flame propagation). The ratio of these components has a significant impact on fuel properties and directly affects the engine efficiency and formation of pollutant emissions. Therefore, we need to have a clear understanding of the chemical-reaction mechanism of the gasoline fuel combustion process. However, studies have shown that an increase in components will lead to an exponential increase in reaction, species, and thermal-physical parameters, and the hundreds of components in gasoline will increase the complexity of the reaction mechanism to an unacceptable level [5]. Therefore, it is a feasible research trend to describe the physical and chemical properties of gasoline fuel with one or more components; that is, to find gasoline surrogate fuels to replace gasoline for chemical kinetic-model research. A good gasoline surrogate fuel and chemical kinetic model can not only accurately simulate the properties of real gasoline but also greatly reduce the difficulty of experimental research and the computational cost of numerical simulation.

Gasoline surrogate fuels are composed of a limited number of components. A binary mixture of isooctane and n-heptane (called primary reference fuel, PRF) is commonly used to match a given research octane number or motor octane number (RON or MON) [6,7,8]. The RON of PRF surrogate fuels is equal to MON. However, the RON and MON of actual gasoline are not the same, generally RON > MON. During gasoline combustion, the octane sensitivity (S, S = RON − MON) and antiknock index (AKI, AKI = RON + MON/2 [9]) have an impact on engine performance, power, and efficiency. Therefore, more components are needed to match the various properties of the actual gasoline. Since they are known to have high octane numbers and high sensitivity, aromatics are widely used to improve octane in gasoline [10]. Toluene reference fuels (TRFs), consisting of toluene, n-heptane, and isooctane, have been studied extensively [11,12,13,14]. Andrae et al. [11] constructed a simplified mechanism for TRF with 137 species and 633 reactions, and this mechanism has good predictive performance in terms of ignition-delay times. Badra et al. [12] reported a correlation between the octane number of a TRF surrogate and the ignition time of a homogeneous gas-phase air fuel.

Mixtures with more than three typical components (TRF) are called multicomponent gasoline surrogate fuels. Such a mixture usually includes other components required to reproduce the real gasoline characteristics in addition to the three typical components. More components need to be added to the surrogate fuel to match important characteristics such as gasoline functional group or hydrocarbon distribution, average molecular weight, and octane number sensitivity, which are not met by PRF and TRF. Therefore, surrogate fuel should include components that can cover a wider range of typical gasoline. Real gasoline usually contains cycloalkanes, olefins, and oxygenates. The typical chemical kinetic mechanisms of four-component and five-component gasoline surrogate fuels are mainly linear alkanes, branched alkanes, aromatic hydrocarbons, olefins, and oxygen-containing components. Liang [15] added diisobutylene (DIB) as a representative of olefins in gasoline to TRF and developed a four-component simplified mechanism suitable for homogeneous charge compression ignition (HCCI) engines. Shi [16] et al. developed an oxygenated gasoline surrogate model based on the effect of water addition on chemical kinetics. In order to improve the explosion resistance of gasoline, Andrae [17] developed a five-component skeleton mechanism (including isooctane, n-heptane, toluene, diisobutylene, and ethanol) by adding ethanol to gasoline surrogate fuels. The establishment of these models can further understand the effects of different components on the combustion characteristics of gasoline (such as ignition delay and laminar flame speed).

Cycloalkanes are an important component of transportation fuels, with different percentages of about 10% and 40% in gasoline and diesel [10,18]. And among all cycloalkanes, cyclohexane (CHX) has been more studied due to its simple ring structure [19,20,21,22,23]. Daley et al. [19] observed that the autoignition of CHX under stoichiometric conditions was more dependent than olefins on pressure. An experimental study on the autoignition behavior of CHX in a rapid compression machine (RCM) was carried out by Vranckx [21], revealing a very strongly pressure-dependent negative temperature coefficient (NTC) behavior within the 710–870 K temperature regime. Silke et al. [24] developed a detailed chemical kinetic model for simulating cyclohexane oxidation in a fast compressor and jet-stirred reactor, which reproduced well the overall reactivity of cyclohexane oxidation and many of the experimental data on the distribution of intermediate species, but the model was unable to reproduce the experimentally observed amount of 1,2-epoxycyclohexane.

In order to make the physical and chemical properties of gasoline surrogate fuels closer to those of actual gasoline or to better analyze the mechanism of carbon soot formation, some researchers have started to focus on the development of simplified mechanisms for multicomponent gasoline surrogate fuels containing CHX. Li et al. [25] developed a five-component gasoline surrogate fuel kinetic model with 563 species and 2915 reactions, which consisted of n-heptane, isooctane, toluene, diisobutene, and cyclohexane. This five-component model can predict ignition-delay times well, but the excessive number of reactions makes it difficult to cover a wider range of fuel properties (e.g., laminar flame speed). The literature also does not study the effects of single components (e.g., cyclohexane) on surrogate fuel. A reduced 11-component (n-heptane, iso-octane, toluene, ethanol, methanol, n-decane, n-dodecane, n-hexadecane, diisobutylene, cyclohexane, and methyl-cyclohexane) chemical mechanism consisting of 178 species and 758 reactions is proposed by Ren et al. [26] for combustion and soot formation predictions of wide distillation fuel.

Although the addition of cyclohexane to gasoline surrogate fuels has been well studied, few studies have analyzed the effect of cyclohexane on the combustion characteristics of gasoline surrogate fuels. However, cyclohexane content affects the ignition quality of gasoline engines as well as the formation of polycyclic aromatic hydrocarbons (PAHs) and carbon soot. In addition, Andrae [27], in his previous detailed chemical kinetic modeling, showed that there is kinetic coupling between aromatic hydrocarbons and the main reference fuel, which affects the number and type of radicals and thus controls the ignition and combustion of the fuel. A study by Sarathy [28] found a similar coupling between cycloalkanes and aromatic hydrocarbons. Therefore, it is necessary to analyze the effect of cyclohexane on surrogate fuels in multicomponent gasoline surrogate fuels containing cyclohexane. In the study of RCCI combustion characteristics, Raza [29] found that toluene and cyclohexane inhibited the reactivity of multicomponent surrogate fuels, and they also controlled the low-temperature heat-release rate and increased the high-temperature heat-release rate.

The above studies have shown that cyclohexane components have a notable influence on the ignition and combustion processes of gasoline. However, in the development of gasoline fuel models, fewer studies have analyzed the effect of the addition of the new species (cyclohexane) on the original components and their predicted performance. In this study, the effect of adding cyclohexane to a four-component mixture consisting of n-heptane, isooctane, toluene, and diisobutene on the combustion of the surrogate fuel was investigated by kinetic-model simulation studies using a preconstructed simplified mechanism [30] for a five-component gasoline surrogate fuel containing cyclohexane. The predictive performance of ignition-delay time, laminar flame speed, and HCCI cylinder pressure curves of three-, four- and five-component gasoline surrogate fuels was first explored against real gasoline. Then, the effect of cyclohexane addition on the ignition and combustion performance of the surrogate fuels was analyzed by adding different percentages of cyclohexane to the four-component surrogate fuels formulated by Fikri [31]. The aim was to investigate the effect of the addition of a new species (cyclohexane in this paper) on the original components and their predicted performance during model development.

## 2. Real Gasoline Predictive Performance Analysis of Simplified Mechanism

The model used in this study is a five-component gasoline surrogate fuel kinetic model (CHX-DIB-Toluene Reference Fuel, CDTRF) containing n-heptane, isooctane, toluene, diisobutylene, and cyclohexane, which we constructed in our previous work [30]. In our previous work, we added a simplified cyclohexane kinetic model to a four-component gasoline surrogate kinetic model (DIB-Toluene Reference Fuel, DTRF). A simplified five-component mechanism for gasoline applicable to thin combustion was obtained, and the constructed model was evaluated in terms of ignition-delay time, laminar flame speed, and species-distribution data.

In this section, the predictions of ignition-delay times, laminar flame speed, and HCCI cylinder pressure curves for three-, four-, and five-component gasoline surrogate fuels are explored, and the ignition and combustion behaviors of the surrogate fuels when the components are varied are verified against real gasoline, respectively.

### 2.1. Calculation of Physical Parameters

Based on the literature data [9,32,33], the physicochemical properties such as RON, MON, calorific value, H/C, and molecular weight of the five components of n-heptane, isooctane, toluene, diisobutene, and cyclohexane were compiled, as shown in Table 1.

Real gasoline with different physicochemical properties can be described to some extent by adjusting the composition ratio between the five components. Over the years, the development of gasoline surrogate fuels has prompted many researchers to develop methods for formulating gasoline surrogate fuels [9,34,35,36] with the aim of matching a wider range of physicochemical properties of gasoline. Morgan et al. [35] proposed a computational model for the nonlinear mixing of component volume fractions, which is a second-order modified nonlinear model that uses experimentally determined TRF octane values to accurately predict the octane values of other arbitrary TRF fuels. Knop et al. [34] used a simple-component molar fraction linear mixing calculation method to predict the physical and chemical properties of blended fuels based on the physical and chemical properties of the pure components of the fuel, which compares well with the equation in the literature [36]. In the recent work of Del Pecchia [9], fuel combustion properties (e.g., ignition-delay time and laminar flame speed) were also taken into account in the formulation of surrogate-fuel components. Although Morgan et al.’s method more accurately matches the physical and chemical properties of three-component surrogate fuels, it is difficult to apply to more complex species distributions (four- and five-component). Therefore, the method of linear mixing of component molar fractions was chosen for the calculation of gasoline surrogate fuel component proportions in this paper. The RON, MON, calorific value (LHV), density, H/C, and molecular weight of the surrogate fuels were calculated by the following equations:

(1) $$\sum_{i=1}^{n}x_{i}RON_{i}=RON_{blend}$$

(2) $$\sum_{i=1}^{n}x_{i}MON_{i}=MON_{blend}$$

(3) $$\sum_{i=1}^{n}x_{i}LHV_{i}=LHV_{blend}$$

(4) $$\frac{\sum_{i=1}^{n}x_{i}M_{i}}{\sum_{i=1}^{n}x_{i}\frac{M_{i}}{\rho_{i}}}=\rho_{blennd}$$

(5) $$\frac{\sum_{i=1}^{n}x_{i}H_{i}}{\sum_{i=1}^{n}x_{i}H_{i}}=\frac{H}{C}$$

Here, *i* and n denote the *i*-th component and the total group fraction in the compound, respectively; $x_{i}$, $RON_{i}$, $MON_{i}$, $LHV_{i}$, $M_{i}$, $\rho_{i}$, $H_{i}$, and $C_{i}$ denote the molar fraction, research octane number, motor octane number, low calorific value, molecular weight, density, number of H atoms, and number of C atoms of component *i*. It should be noted that the sum of the molar fractions of the components is one in the calculation of each physical property, i.e.,

(6) $$\sum_{i=1}^{n}x_{i}=1$$

The basic physical and chemical properties (density, molecular weight, H/C, RON, MON, etc.) of the surrogate fuels can be basically determined by the above formulae to match the appropriate surrogate to the actual gasoline fuel. Therefore, the proportion of surrogate components is different for different actual gasoline, and even if two actual fuels have similar ignition and flame-combustion characteristics, their surrogate components are not the same.

### 2.2. Ignition Performance Analysis of Surrogate

Sarathy et al. [28] measured the ignition-delay time of FACE F and FACE G gasolines from Saudi Aramco in 2 MPa and 4 MPa shock tubes and rapid compression machines. Their study showed that the gasoline of FACE F with lower octane sensitivity exhibited a smaller slope in the negative temperature coefficient region, as fuels with lower octane sensitivity behaved more like n-alkanes and showed a distinct NTC characteristic [37]. In addition, they found that the ignition-delay time of lower-octane sensitive gasoline (e.g., FACE F, S = 5.6) could be captured by simple binary PRF or ternary TRF mixtures. However, the ignition behavior of higher-octane sensitive gasolines (e.g., FACE G, S = 11) requires more complex multicomponent surrogate mixtures. Therefore, in this paper, three surrogate fuels are formulated for the hydrocarbon ratio composition of FACE G gasoline (7.9% n-alkanes, 38.3% isoparaffins, 14.1% cycloalkanes, 31.8% aromatics, and 7.9% olefins in molar fractions), combining existing formulations in the literature and the computational work in this paper, as shown in Table 2.

**Figure 1:**
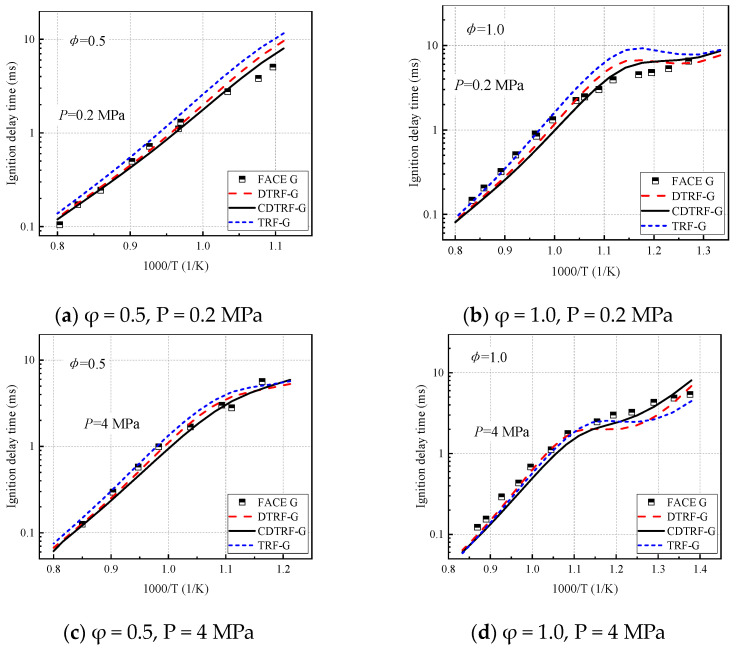
Simulation of ignition-delay time of FACE G gasoline shock tube with three surrogate mixtures.

Figure 1 shows the capture of FACE G gasoline shock tube ignition-delay time data by TRF-G, DTRF-G, and CDTRF-G. Simulations are performed at two different equivalence ratios of lean conditions (φ = 0.5) and chemical equivalence ratio (φ = 1.0) and at pressures of 2 MPa and 4 MPa. As can be seen from the figure, the data are well predicted for various surrogate mixtures above 1000 K (1000/T < 1.0) at all pressures. The behavior of all surrogate fuels (TRF-G, DTRF-G, and CDTRF-G) is similar in these high-temperature regions, where the ignition-delay time decreases with increasing temperatures. Nevertheless, above 1000 K, the simulations for CDTRF-G under lean conditions are quantitatively closer to the measured data, while TRF-G is more dominant at φ = 1.0, with negligible differences between the three. Below 1000 K, the best predictions are obtained for the five-component mixture CDTRF-G, although below 900 K (1000/T > 1.1) the three surrogate-mixtures simulations all reproduced the NTC behavior. However, only the CDTRF-G surrogate fuel accurately captured the ignition-delay data. This suggests that the addition of cyclohexane causes the fuel to experience a smaller NTC effect on the combustion behavior. In addition, the variation of the equivalence ratios shows that the newly constructed model has higher accuracy in predicting real gasoline under lean conditions (φ = 0.5), while the accuracy decreases at chemical-equivalence ratios, which is due to the fact that the model construction and validation process is performed based on lean combustion and the model has better prediction performance for combustion under lean conditions.

### 2.3. Flame Propagation Performance Analysis of Surrogate Fuel

During previous validation of chemical kinetic models, the dependence of laminar flame speed on fuel combustion temperature and pressure was observed. The laminar flame speed increased with the increase in temperature and decreased with the increase in pressure [30]. However, the effect of different fuel components on the laminar flame speed was not considered. Sileghem [38] used the heat-flux method on a flat flame adiabatic burner to measure the laminar flame speed of Exxon 708629-60 gasoline from ExxonMobil, Irving, TX, USA, which consists of 10.37% n-alkanes, 40.2% isoalkanes, 34.39% aromatics, 9.39% cycloalkanes, and 5.65% olefins by volume fraction. In their study, a mixture of 1/3 isooctane, 1/3 n-heptane, and 1/3 toluene by volume fraction was used to predict the laminar flame speed and showed good consistency. In addition, Sileghem [38] did not give the relevant physicochemical properties (e.g., RON, MON) of Exxon 708629-60 gasoline. Therefore, in this work, in addition to using their formulated TRF-Exxon fuel and the DTRF-Exxon fuel given by Fikri [31], the CDTRF-Exxon surrogate prepared according to Exxon 708629-60 gasoline volume fraction is also used to predict laminar flame speed, as shown in Table 3.

**Figure 2:**
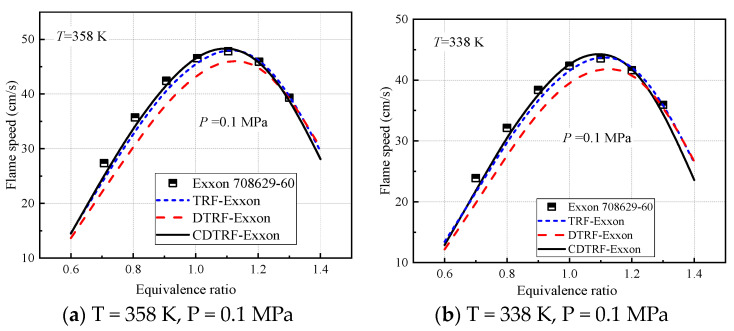
Three surrogate mixtures simulate the laminar flame speed of Exxon 708629-60 gasoline.

The prediction of laminar flame speed by three surrogate fuels is shown in Figure 2. It can be seen from Figure 2 that the TRF-Exxon surrogate mixture of 1/3 isooctane, 1/3 n-heptane, and 1/3 toluene volume ratio calculated by this model can match the laminar flame speed of gasoline. The prediction performance of DTRF-Exxon is the worst due to the fact that the four-component fuel components contain too much toluene volume fraction, which reduces the reactivity of the fuel and shortens the laminar flame speed near the lean combustion and even the chemical-equivalence ratio. The calculation results of the CDTRF-Exxon surrogate mixture are consistent with those of TRF-Exxon and show higher accuracy in lean combustion (φ < 1.0). According to the explanation of Sileghem [38], although the laminar flame speed of TRF-Exxon, the toluene reference fuel, is consistent with that of Exxon 708629-60 gasoline, the mixture of isooctane, n-heptane, and toluene will not be able to predict the experimental data of ignition, combustion, or engine characteristics of all fuels (ignition-delay time, evaporation characteristics, emissions, etc.). Therefore, the CDTRF-Exxon with more components may be used to simulate specific engine combustion properties.

### 2.4. HCCI Engine Combustion-Performance Analysis of Surrogate

To further evaluate the effectiveness of the chemical kinetic model in simulating the combustion characteristics of gasoline fuels when different surrogate-fuel mixture compositions are selected, a homogeneous reactor HCCI model is used to simulate the variation of pressure curves of surrogate fuels operating in an HCCI engine. Christensen [39] measured the cylinder pressure curves of typical high-octane gasoline (Grön 98 MK1, RON = 98.5, MON = 88) on the Volvo TD100 engine. The engine size is shown in Table 4, and the intake conditions are shown in Table 5 for three cases of engine-compression ratio and intake temperature changes.

The fuel used is a high-octane gasoline (Grön 98 MK1) with a RON of 98.5 and a MON of 88. Equations (1) and (2) are used to calculate the RON and MON for the three Grön 98 MK1 gasoline surrogate mixtures. The composition of the fuel is shown in Table 6.

The in-cylinder pressures of HCCI engines are predicted for the three surrogate fuels using the selected CDTRF kinetic model (equivalence ratio 0.333). Considering the uncertainty of the energy conversion of the fuel–air mixture from the engine intake to the time of entering the intake valve, the starting moment of the calculation is chosen to be −30 °CA (the upper dead point is 0 °CA). According to the experimental data, the initial conditions for the calculation at the moment of −30 °CA are condition 1: temperature 730 K, pressure 1.4 MPa; condition 2: temperature 750 K, pressure 1.35 MPa; condition 3: temperature 770 K, pressure 1.3 MPa.

**Figure 3:**
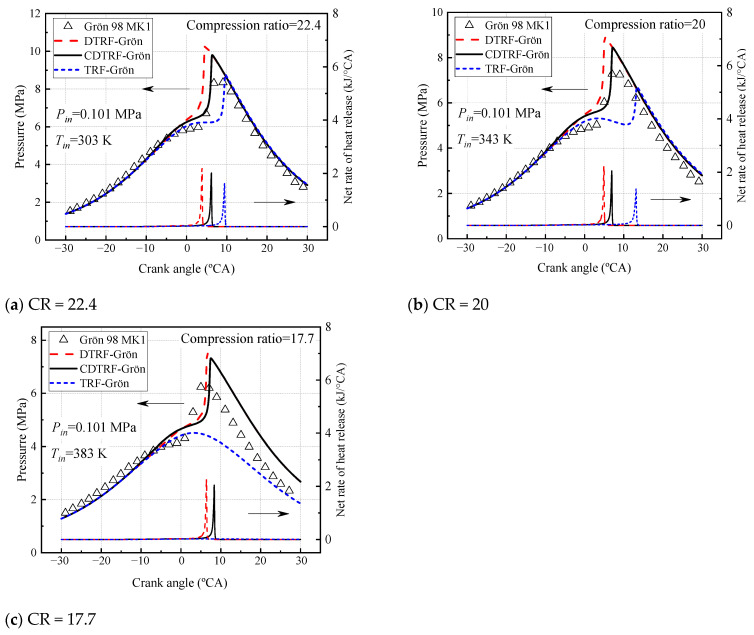
HCCI engine experiments and simulated pressure curves of gasoline and its three surrogate mixtures under different compression ratios.

Figure 3 shows the comparison between the experimental pressure curves of the engine and the pressure curves calculated from the surrogate fuels in Table 6. It can be seen that in all three cases, the DTRF-Grön and CDTRF-Grön surrogate fuels accurately capture the sudden pressure change after the compression top dead center (TDC), which means that heat is released at this moment. In contrast, TRF-Grön fuel is too resistant to spontaneous combustion under these specific engine conditions. When the compression ratio is reduced, the ignition of TRF-Grön is delayed (CR = 20), and it is even difficult to spontaneously ignite (CR = 17.7). The ignition times of DTRF-Grön and CDTRF-Grön surrogate fuels are similar in all three cases. According to the analysis of the fuel components, it is difficult for TRF-Grön to self-ignite at the compression ratio of 17.7 because it contains too many toluene components, and DTRF-Grön and CDTRF-Grön can self-ignite not because of the addition of new substances to enhance the reactivity, but because of the composition of a variety of components to reduce the use of toluene, and then achieve ignition near TDC under the same conditions. In addition, since the adiabatic model is used in HCCI simulation, the in-cylinder pressure calculated by all models is higher than the experimental value at the ignition timing. 

In addition, the accuracy of the model is not only to predict the cylinder pressure but also to be able to accurately predict the combustion exothermic rate. The calculation results of the exothermic rate of three surrogate fuels are shown in Figure 3 and it can be seen that the exothermic rate curves and peak positions of the surrogates with different compositions are different. In fact, during engine operation, the in-cylinder combustion heat-release rate can be calculated by calculating the value of the variation of the in-cylinder pressure with the crankshaft rotation angle of the engine. As can be seen in Figure 3, the peak magnitude of the exothermic rate of the three surrogate fuels is proportional to their cylinder pressure curves in the engine, and the peak exothermic rate occurs at the peak pressure position. In addition, it can be seen that the exothermic rate of TRF-Grön is almost 0 kJ/°CA in the calculated crankshaft angle range at a compression ratio of 17.7. Therefore, there is no sudden change in pressure and thus it fails to ignite.

## 3. Effect of Cyclohexane on Combustion Characteristics of Gasoline Surrogate Fuel

In the previous section, the ignition and combustion characteristics of three-, four-, and five-component gasoline surrogate fuels were analyzed with reference to experimental data on the ignition, flame propagation, and HCCI in-cylinder combustion of common gasoline on the market. However, the development of the model needs to consider the effect of the addition of new species on the original components and their predicted performance. In this section, the effect of adding cyclohexane to a four-component mixture consisting of n-heptane, isooctane, toluene, and diisobutene on the combustion of the surrogate fuels is explored through kinetic-model simulation studies. This four-component surrogate fuel was formulated by Fikri et al. [31], and the physical parameters of the five-component surrogate fuel mixture after the addition of cyclohexane were calculated from Equations (1)–(5). They have a similar low calorific value (42.4 kJ/kg), but their RON is decreasing with increasing the proportion of cyclohexane, as shown in Table 7.

For the fuels formulated in Table 7, the ignition-delay times calculated in the zero-dimensional homogeneous reactor and the laminar flame speed calculated in the one-dimensional flame reactor are compared separately, and kinetic analysis is performed to interpret the results. Finally, two of the surrogate fuels are simulated using an HCCI homogeneous reactor. The effects of surrogate-fuel component changes, engine speed, and intake air temperature on fuel autoignition are studied and explained from a fuel-chemistry perspective.

### 3.1. Effect of Cyclohexane on Ignition-Delay Time

The ignition characteristics of the fuel are controlled by the chemical-reaction kinetic process of the fuel itself and the input of external boundary conditions. Therefore, in this paper, the effect of cyclohexane addition on the ignition-delay time of gasoline–surrogate fuel mixtures are first investigated under lean combustion conditions.

**Figure 4:**
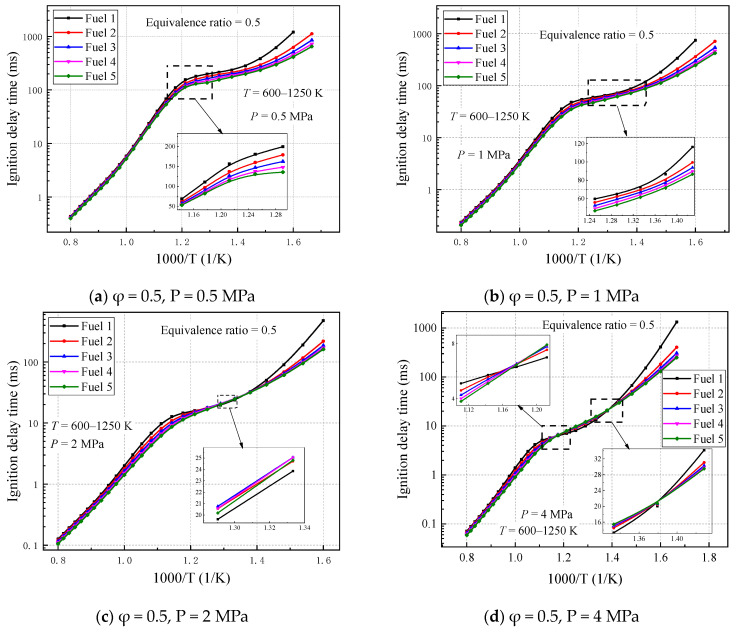
Ignition-delay time of fuels 1–5 under different pressures, equivalence ratio of 0.5, and temperature range of 600–1250 K.

The ignition-delay times are calculated for five gasoline–surrogate fuel mixtures (Fuel 1–5) under the lean combustion condition (equivalent ratio of 0.5), temperature range of 600–1250 K, and pressure range of 0.5–4 MPa. The ignition-delay times of the five surrogate-fuel models calculated using the selected mechanism are given in Figure 4.

As can be seen in Figure 4, at higher initial temperatures (T > 1000 K), the differences in ignition-delay times of the five fuels are not significant and maintained a consistent trend with temperature changes. However, at higher pressures (2 MPa or 4 MPa), the differences in ignition-delay times of the five fuels increase, and the more surrogate fuels containing cyclohexane content, the smaller the ignition-delay times. When the initial temperature is small (T < 715 K), the ignition-delay times of the five surrogate fuels are gradually separated, with fuel 1, which does not contain cyclohexane, being the most affected by the temperature, indicating that the addition of cyclohexane reduces the ignition delays of the surrogate fuels in the low-temperature reaction stage. The largest difference occurs in the initial temperature range of 715–1000 K. At lower pressures, fuel 1 without cyclohexane still lags behind the other fuels. As the pressure increases, the ignition-delay times of fuels 2–5 are relatively higher than those of fuel 1, with fuel 5, which contains the largest volume fraction of cyclohexane, exhibiting the least pronounced NTC (Figure 4d), suggesting that the addition of cyclohexane suppresses the negative temperature behavior of the surrogate fuels in the midtemperature region and becomes more pronounced as the pressure increases.

**Figure 5:**
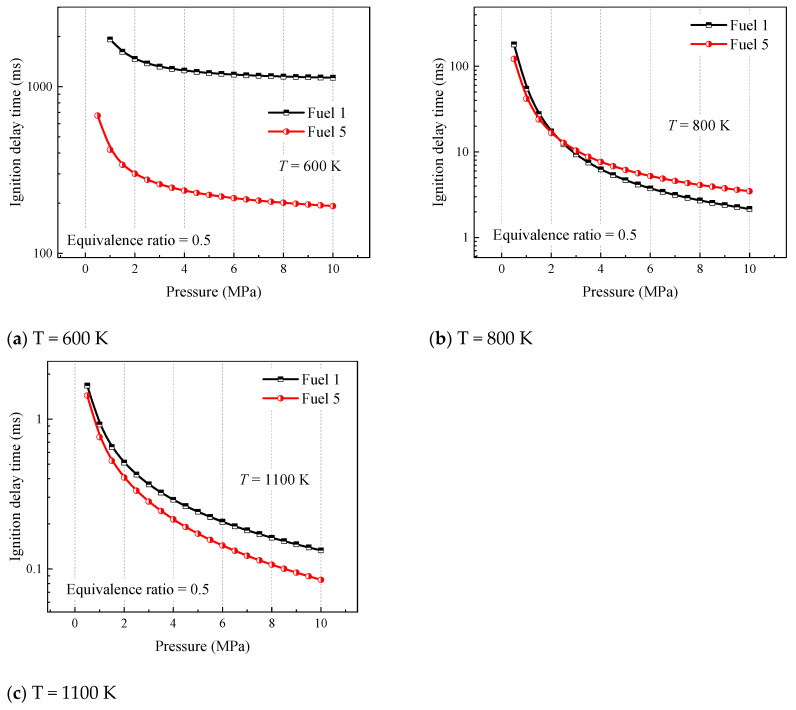
Ignition-delay time of fuels 1 and 5 in the pressure range of 1–10 MPa at different temperatures (equivalence ratio of 0.5).

In order to further study the effect of initial pressure on the ignition laws of surrogate fuels, the ignition laws are investigated at different temperatures with different cyclohexane concentrations. Figure 5 analyzes the ignition of fuel 1 and fuel 5 at initial pressures of 1–10 MPa, where fuel 1 is a four-component surrogate fuel without cyclohexane and fuel 5 is the surrogate fuel with the largest volume fraction of cyclohexane constructed in this paper. Three representative temperatures are selected according to Figure 5, 600 K for the low-temperature region, 800 K for the medium-temperature region, and 1100 K for the high-temperature region.

The results shown in Figure 5 indicate that the difference in ignition-delay time between fuel 1 and fuel 5 at 600 K and 1100 K increases with increasing pressure, which explains the phenomenon in Figure 4 that the difference in ignition-delay time between different fuels at low and high temperatures increases with increasing pressure. In addition, the gap in ignition-delay time between the two surrogate mixtures is the largest at 600 K. Based on the previous analysis, this is because the chemical reaction rate in the low-temperature phase is more dependent on the oxidation of the fuel macromolecules. At 800 K, it can be seen that the ignition-delay time of fuel 1 is not always higher than that of fuel 5, and both shift at a pressure of 2.25 MPa, as shown in Figure 5b. It can be seen that the addition of cyclohexane changes the ignition pattern of the four-component surrogate fuels in the midtemperature region. The ignition delay of the fuel containing cyclohexane in the midtemperature phase is less dependent on pressure, which affects the NTC behavior of the surrogate fuel in this region.

The root causes of the different surrogate fuels showing different ignition patterns are further analyzed at the microscopic level by chemical kinetics. For the two fuels (fuel 1 and fuel 5) with the largest difference in cyclohexane content, sensitivity analyses are performed at low (690 K), medium (800 K), and high (1200 K) temperatures at an initial pressure of 2 MPa and an equivalence ratio of 0.5, and the top ten most-sensitive reactions are selected.

**Figure 6:**
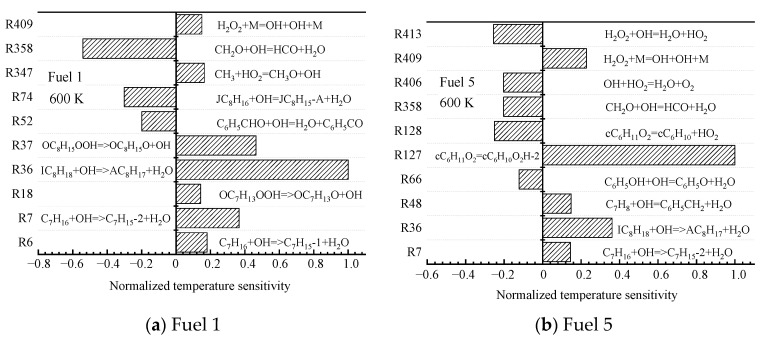
Temperature sensitivity at 690 K.

The sensitivity reactions of the fuel ignition process for fuel 1 and fuel 5 at an initial temperature of 690 K are shown in Figure 6. At low temperatures, the sensitivity of the fuel ignition process is dominated by macromolecular reactions.

R7. C_7_H_16_ + OH => C_7_H_15_-2 + H_2_O

R36. IC_8_H_18_ + OH => AC_8_H_17_ + H_2_O

R37. OC_8_H_15_OOH => OC_8_H_15_O + OH

R127. cC_6_H_11_O_2_ = cC_6_H_10_O_2_H-2

R358. CH_2_O + OH = HCO + H_2_O

Among them, for fuel 1, R7 and R36 are the reactions of straight and heterochain alkanes with OH radicals to remove a hydrogen atom to form the corresponding alkyl radical, and R37 is the further decomposition of hydroperoxyalkyl radicals (QOOH); for fuel 5, R36 is the same as the dehydrogenation of chain alkanes, and R127 is the isomerization of cyclohexane oxide cC_6_H_12_O_2_, which in fuel 5, the surrogate-fuel ignition process showed higher sensitivity; in addition, the small molecule reaction R358. CH_2_O + OH = HCO + H_2_O exhibits a greater sensitivity during fuel 1 ignition, which indicates that the depletion of OH radicals in the R358 reaction reduces the reactivity of fuel 1 fuel. The above analysis illustrates that the fuel ignition process in the low-temperature region is controlled to a great extent by the initial oxidation and dehydrogenation reactions of fuel macromolecules, and the cyclohexane molecules of fuel 5 are the first to react, and the accumulation of OH radicals drives the early ignition of fuel 5.

**Figure 7:**
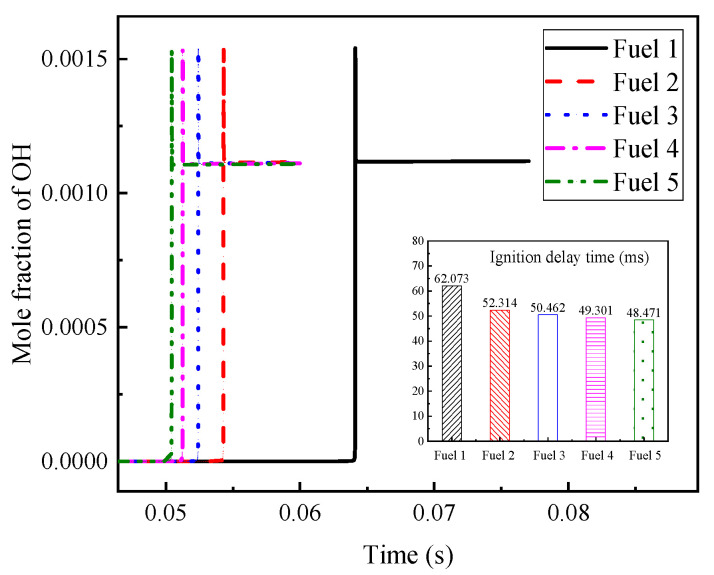
OH mole fraction and corresponding ignition-delay time of five surrogate fuels at low temperature 690 K.

Among the above reactions with large, normalized sensitivity coefficients, OH radicals are found to appear more often, which indicates that OH radicals have a greater influence on the ignition and combustion of the system. The OH radical molar fraction curves for the fuels in Table 7 are plotted in Figure 7 at an initial temperature of 690 K, an initial pressure of 2 MPa, and an equivalence ratio of 0.5, and the corresponding ignition-delay times of the fuels are compared in the figure. The peak appearance of OH radicals for fuel 1 fuel is different from the other four fuels by about 10 ms, and the final OH radical production for the five fuels remained at a consistent level. The corresponding ignition-delay times indicate that the peak moments of OH radicals can correspond to the ignition-delay times of their fuels one by one.

**Figure 8:**
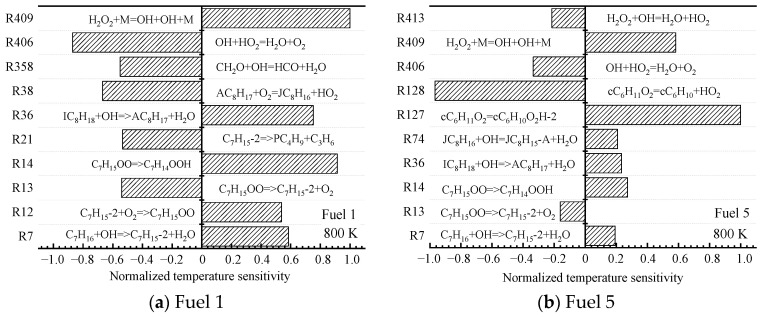
Temperature sensitivity at 800 K.

Next, the sensitivity of the ignition characteristics of fuel 1 and fuel 5 at a medium temperature of 800 K is analyzed. Unlike the low-temperature region, the temperature sensitivities calculated for the two surrogate-fuel ratios in the medium-temperature region appear significantly different. This is shown in Figure 8.

R14. C_7_H_15_OO => C_7_H_14_OOH

R36. IC_8_H_18_ + OH => AC_8_H_17_ + H_2_O

R38. AC_8_H_17_ + O_2_ = JC_8_H_16_ + HO_2_

R127. cC_6_H_11_O_2_ = cC_6_H_10_O_2_H-2

R128. cC_6_H_11_O_2_ = cC_6_H_10_ + HO_2_

R358. CH_2_O + OH = HCO + H_2_O

R406. OH + HO_2_ = H_2_O + O_2_

R409. H_2_O_2_ + M = OH + OH + M

For fuel 1, the reactions with larger normalized sensitivity coefficients shift from macromolecular reactions to small molecular reactions (R358, R406, and R409), and the small molecular reaction R409 has the largest normalized sensitivity coefficient. H_2_O_2_ is decomposed into two OH radicals under the action of the third body, which increases the system temperature and reduces the ignition-delay time, leading to the formation of the NTC region of the fuel. However, the reactivity of fuel 1 in the middle-temperature region is still lower than that in the high-temperature region, because some macromolecular reactions (R14, R36, and R38) have large, normalized sensitivity coefficients. From Figure 8b, it can be seen that the fuel sensitivity of fuel 5 is still dominated by macromolecular reactions (R127 and R128), which are isomerization and decomposition reactions of cyclohexane oxide cC_6_H_12_O_2_, respectively. In contrast, the small molecular reaction R409 only occupies a small proportion. This explains the phenomenon that fuel 2~5 inhibit the NTC effect in Figure 4d.

**Figure 9:**
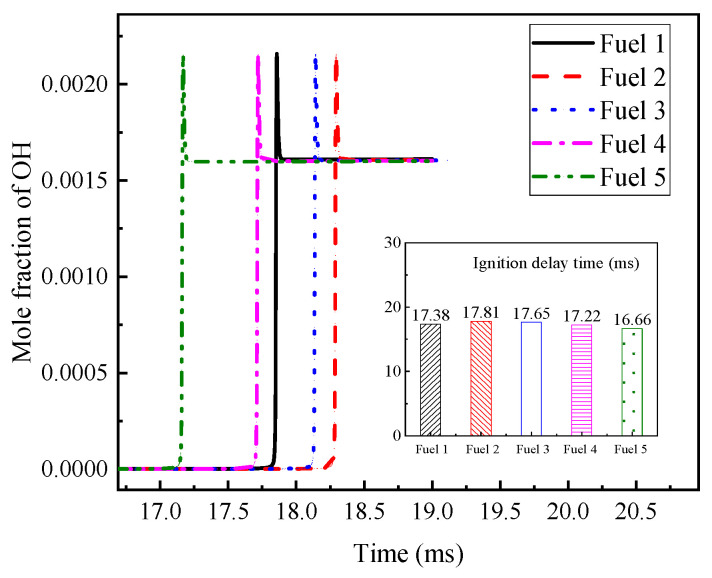
OH mole fraction and corresponding ignition-delay time of five surrogate fuels at medium temperature 800 K.

The OH radical generation curves for the five surrogate fuels in Table 7 are calculated. The calculation conditions are 800 K initial temperature, 2 MPa pressure, and 0.5 equivalence ratio. Figure 9 gives the OH radical generation curves and the corresponding ignition-delay times for the five surrogate fuels. It can be seen that the OH radical molar fraction of the five surrogate fuels peaks at 17–18.5 ms, and the OH radical peak of fuel 1 peaks earlier than that of fuel 2 and fuel 3, while the ignition-delay time of fuel 1 decreases correspondingly. This fully illustrates that the addition of cyclohexane has a great influence on the fuel NTC effect.

**Figure 10:**
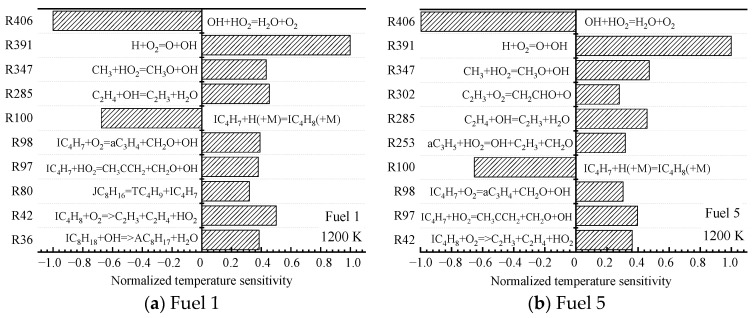
Temperature sensitivity at 1200 K.

Finally, the sensitivities of the ignition characteristics of fuel 1 and fuel 5 at high temperatures of 1200 K are analyzed. Comparing the high-temperature normalized sensitivity coefficients in Figure 10a,b, it can be seen that fuel 1 and fuel 5 basically contain the same high-temperature sensitivity responses, and although the top ten responses of sensitivity are not identical, several important primitive responses of greater sensitivity are consistent.

R100. IC_4_H_7_ + H(+M) = IC_4_H_8_(+M)

R391. H + O_2_ = O + OH

R406. OH + HO_2_ = H2O + O_2_

Among them, the isobutylene radical hydrogenation reaction represented by the radical reaction R100 has a large, negative temperature-sensitivity coefficient, and the consumption of H radicals suppresses the elevation of the system temperature. In addition to exhibiting a large negative temperature sensitivity coefficient is the radical reaction R406, and the absolute value of the sensitivity coefficient is the largest for this radical reaction, which is a depletion reaction of OH radicals, and the unstable intermediate product, peroxyhydroxyl radical HO_2_, generates water and oxygen molecules with OH radicals. Although the depletion of the active molecule OH radical has an inhibitory effect on the system ignition, the oxygen molecules generated by the reaction provide the oxidant for the subsequent fuel reaction. The radical reaction R391 shows the greatest positive sensitivity, which is a branching reaction of the chain, consisting of H radicals colliding with O_2_ molecules to generate active OH radicals and O radicals, while O radicals continue to collide with H_2_ molecules parametrically OH radicals, increasing the concentration of OH radicals in the system, raising the system temperature and prompting the fuel to reach ignition in a short time.

The small difference in ignition-delay time between the two fuels at high temperatures is mainly attributed to the same reactivity of the small molecule reactions controlling the reaction progression. It can also be seen from Figure 10 that the earlier ignition of fuel 5 compared to fuel 1 is due to the inclusion of more small molecule reactions in the high-temperature positive sensitivity of Fuel 5.

**Figure 11:**
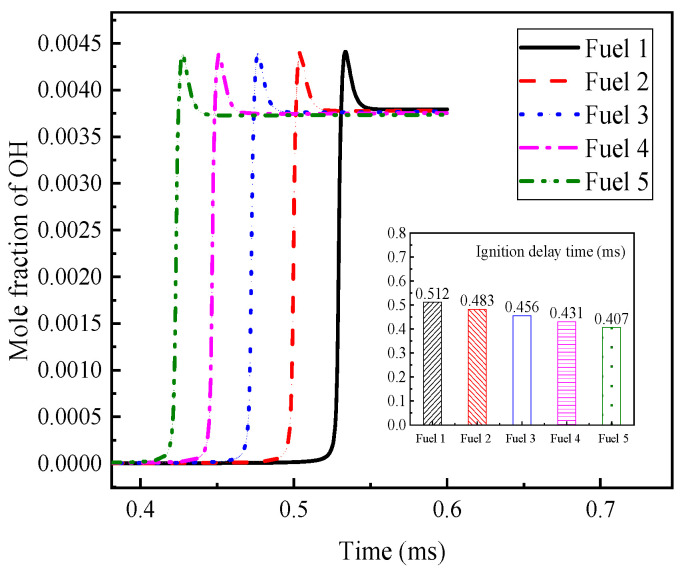
OH mole fraction and corresponding ignition-delay time of five surrogate fuels at high temperature 1200 K.

The OH radical generation curves at high temperatures in Figure 11 again explain the above point, as the OH radical peaks of the five surrogate fuels do not differ by more than 0.1 ms, and the reaction proceeds quite rapidly. This shows that the ignition characteristics of the five surrogate fuels remain the same at high temperatures, with a small difference due to the role of cyclohexane, which is more prevalent at higher concentrations.

The effect of cyclohexane on the ignition-delay time of surrogate fuels was analyzed in the temperature range of 600–1250 K, pressure of 0.1–4 MPa, and lean conditions with an equivalence ratio of 0.5. The addition of cyclohexane inhibits the occurrence of fuel NTC, especially at higher pressures. At low and high temperatures, the addition of cyclohexane shortens the ignition time of the fuel, and this effect is most obvious in the low-temperature region. Based on this influence pattern, the ignition pattern of gasoline containing naphthenic hydrocarbons can be analyzed for the subsequent gasoline containing naphthenic hydrocarbons.

### 3.2. Effect of Cyclohexane on Laminar Flame Speed

As mentioned in the previous validation of the mechanism, applying the chemical kinetic model to the numerical simulation of in-cylinder combustion, besides focusing on the ignition characteristics of the fuel, flame propagation is also an extremely important combustion characteristic, which is related to the normal propagation of the fuel flame during in-cylinder combustion and the study of in-cylinder detonation problems. Therefore, this paper investigates the effect of adding cyclohexane to gasoline surrogate fuels on the laminar flame speed of the overall mixture.

Atmospheric pressure and lower initial-temperature conditions are commonly used to study the laminar flame speed of fuels, and an initial temperature of 298 K and an initial pressure of 0.1 MPa are the general conditions under which the laminar flame speed of fuels is studied into the burner, and the experimental data obtained from different laboratories using different experimental methods can be easily compared at this condition. They can also be used as reference values for analyzing the pressure and temperature dependence of laminar and turbulent flames [40]. In addition, the laminar flame speed of the fuel obtained at higher initial temperature and pressure conditions can carry out the combustion of the gasoline when it is ignited before the top dead center of in-cylinder compression, and this initial temperature and pressure will continue to increase for gasoline compression-ignition engines. In this study, the corresponding laminar flame speeds were also calculated in the premixed laminar flame-speed calculation model [41] using surrogate fuels consisting of five different proportions of components, as shown in Table 7. Two calculation conditions were chosen to represent the above two cases: an initial temperature of 298 K and an initial pressure of 0.1 MPa; and an initial temperature of 500 K and an initial pressure of 2 MPa. In addition, two comparison conditions were also used to better compare the effects of temperature and pressure: an initial temperature of 298 K and an initial pressure of 2 MPa; and an initial temperature of 500 K and an initial pressure of 0.1 MPa. The composition of the air is 79% N_2_ and 21% O_2_.

**Figure 12:**
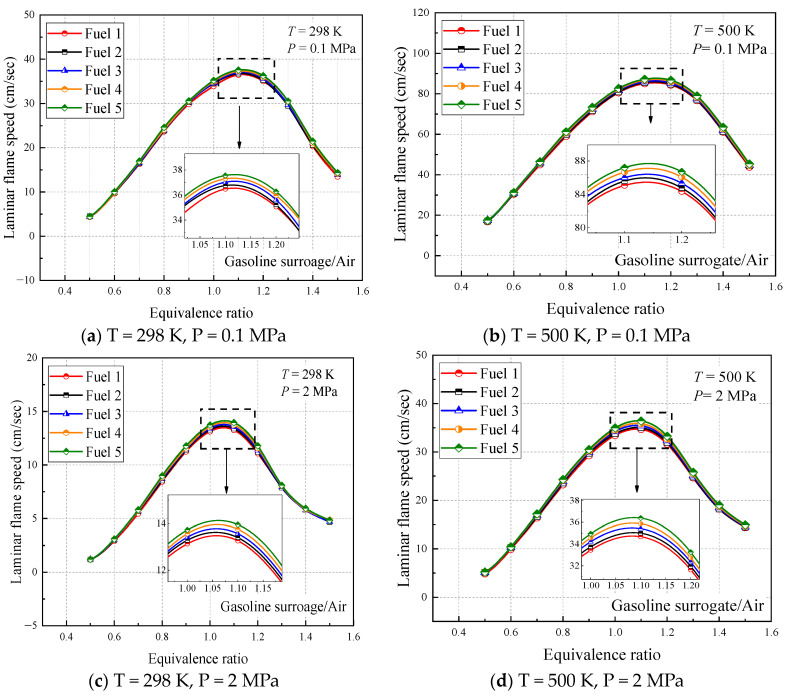
Laminar flame speed of fuels 1–5 in the range of equivalence ratio 0.4–1.5 at different temperatures and pressures.

Figure 12 shows the calculated laminar flame speed for the five surrogate fuel mixtures at four operating conditions. It can be seen that the laminar flame speed of the fuels shows a trend of increasing and then decreasing in covering all the range of equivalent ratios, with the peak occurring near an equivalent ratio of 1.1. This is the same as most of the literature studies [14,38,40,42,43,44], where the chemical-reaction rate is influenced by the fuel-vapor concentration under lean conditions; the higher the equivalent ratio, the higher the laminar flame speed. Under fuel-rich conditions, the chemical reaction rate is mainly affected by the air concentration, and the effect of the equivalent ratio on the laminar flame speed is smaller than that of the specific heat capacity and density of the gas mixture, the larger the equivalent ratio, the smaller the laminar flame speed. The equilibrium between the two is reached near the equivalence ratio of 1.1 to obtain the maximum laminar flame speed. 

From the calculated results of the four working conditions, the trend and magnitude of the laminar flame speed changes are similar for the five surrogate fuels, but by the local enlargement in Figure 12, fuel 5 has the largest calculated result while fuel 1 has the smallest, and this change increases with the comparative increase of cyclohexane in the fuel. This difference can be explained by the fact that the addition of cyclohexane dilutes the proportion of chain alkanes and aromatic hydrocarbons in the mixture. According to the laminar flame speed verification of pure component fuels in previous work [30], the approximate order of magnitude of the laminar flame speed of several components is n-heptane > cyclohexane > isooctane > diisobutene > toluene, which, from a microscopic point of view, is the difference in the concentration of reactive radicals such as H and OH generated by each component. The C-C and C-H bonds in toluene are more stable than the other hydrocarbons, so the addition of cyclohexane increases the laminar flame speed of the fuel mixture, and the small difference is attributed to the proportion of n-heptane, which has a larger flame speed, being reduced accordingly.

The results of the initial temperature and pressure effects on the laminar flame speed can be observed in the calculated results of the four working conditions. From Figure 12a,b, it can be seen that the initial temperature increases from 298 K to 500 K, the laminar flame speed increases significantly, and the location where the peak laminar flame speed appears moves toward the fuel-rich direction, indicating that the laminar flame vs. speed of the fuel with increasing temperature is more dependent on the fuel concentration; while from Figure 12a,c, it can be seen that the initial pressure increases from 0.1 MPa to 2 MPa, the laminar flame speed decreases significantly and the peak laminar flame speed appears in the air-rich direction, indicating that the laminar flame speed of the fuel with increasing pressure is more dependent on the concentration of air.

**Figure 13:**
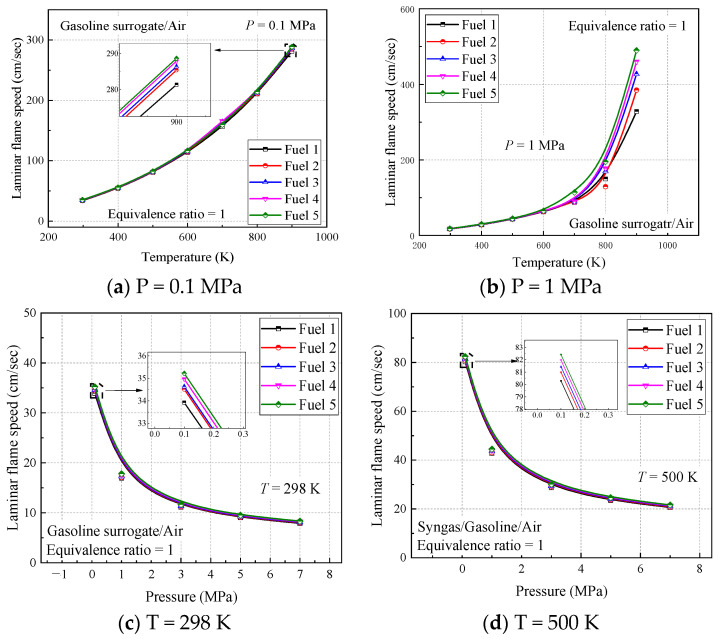
Laminar flame speed of fuels 1–5 in different temperature and pressure ranges when equivalence ratio is 1.

In order to better understand the above phenomenon, the laws of laminar flame speed with temperature and pressure are analyzed in Figure 13, respectively, when the ratio of surrogate-fuel components is varied. The calculated temperature range is 298–900 K and the pressure range is 0.1–7 MPa. From the figures, it can be seen that the laminar flame speed increases with increasing temperature at constant pressure (Figure 13a,b). At a pressure of 1 MPa, the laminar flame speed decreases significantly, but the effect of temperature is also more obvious, and the laminar flame speed increases rapidly after the temperature exceeds 800 K. The effect of fuel components is mainly reflected in the higher-pressure condition (1 MPa), where the simulated flame speed of fuel 1 is the smallest and fuel 5 is the largest. The laminar flame speeds calculated for the five surrogate fuels at an equivalence ratio of one at a constant temperature maintained a consistent trend and decreased with increasing pressure. 

**Figure 14:**
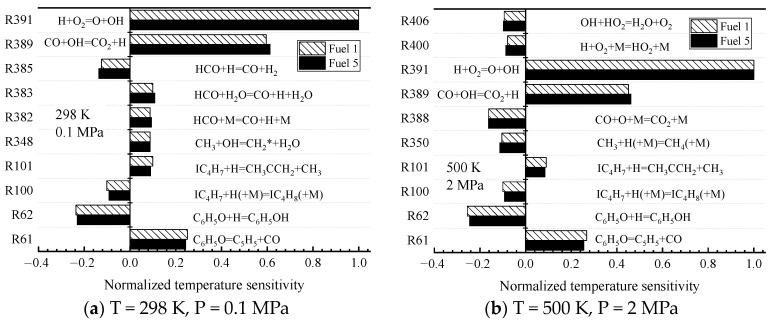
Sensitivity of laminar flame speed under different temperatures and pressures.

The variability of the surrogate-fuel laminar flame speeds was further analyzed in chemical kinetics. The sensitivity of fuel 1 and fuel 5 was calculated in Figure 14 at two operating conditions. Ambient temperature and pressure (298 K, 0.1 MPa) and higher temperature and pressure (500 K, 2 MPa), respectively. The results show that fuel 1 and fuel 5 exhibit the same sensitivity at both operating conditions. The reactions with larger sensitivity coefficients are mainly small molecule reactions.

R62. C_6_H_5_O + H = C_6_H_5_OH

R389. CO + OH = CO_2_ + H

R391. H + O_2_ = O + OH

Among them, R62 exhibits the largest negative sensitivity because the reaction drives the consumption of H radicals. The reaction with the largest positive sensitivity, R391, which was introduced in the previous kinetic analysis of ignition characteristics, generates a large number of active OH radicals and O radicals that initiate the next chain reaction, which greatly increases the chemical reaction rate. R389, which first appeared in the sensitivity analysis, is an oxidation reaction of CO The reaction of CO and OH radicals produces CO_2_ and one H radical, which releases heat and raises the system temperature, and the generated H radicals promote the reaction R391. Therefore, the same sensitivity reaction and sensitivity coefficients of Fuel 1 and Fuel 5 under two working conditions can explain the reason why the laminar flame speed shows the same trend.

Furthermore, all the radical reactions with large sensitivity coefficients in Figure 14 involve the production and consumption of OH and H radicals, suggesting a close association between OH, H radicals, and the laminar flame speed of the fuel. This phenomenon was also reported in a study by Kelley [45], and the similarity of OH and H radicals provides support for the fuel similarity observed in the laminar flame speed. In this study, the similarity between OH and H radical concentrations and laminar flame speed were compared to investigate the relationship between laminar flame speed and OH and H radicals for different component fuels.

**Figure 15:**
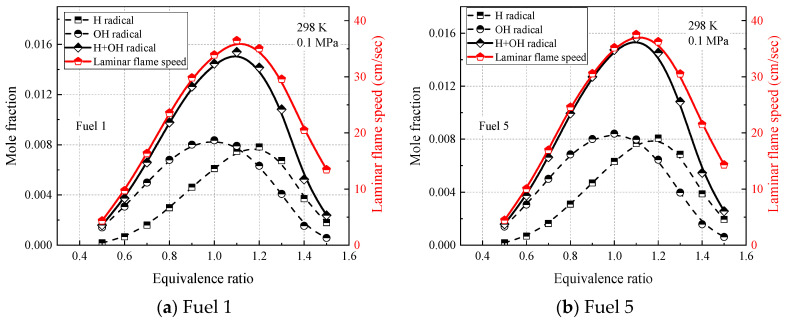
H, OH concentrations and laminar flame speed of fuels 1 and 5 at temperature 298 K, pressure 0.1 MPa, and equivalence ratio 0.5–1.5.

**Figure 16:**
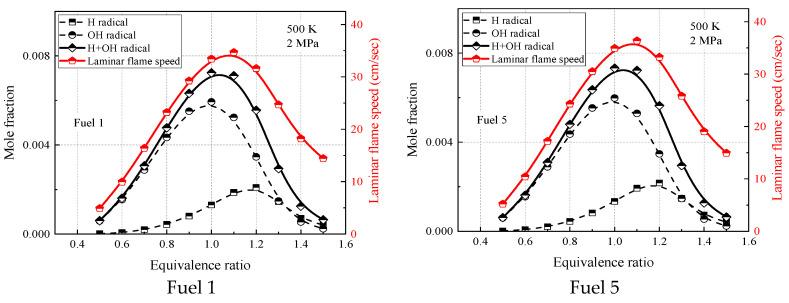
H, OH concentrations and laminar flame speed of fuels 1 and 5 at temperature 500 K, pressure 2 MPa, and equivalence ratio 0.5–1.5.

Figure 15 and Figure 16 show the laminar flame-speed curves and the corresponding OH, H, and OH + H molar fraction curves for fuel 1 and fuel 5 surrogate fuels at two different operating conditions in the range of 0.5–1.5 equivalence ratios, respectively. It is clear that the molar fraction curves of OH radicals or H radicals alone do not show any similarity with the laminar flame speed, while the sum of molar fractions of OH and H radicals and OH + H show consistency with the laminar flame speed in terms of peak equivalence ratio and curve shape. At room temperature and pressure (298 K, 0.1 MPa), the difference between the peak molar fraction of OH radicals and H radicals is not significant, while at higher temperature and pressure (500 K, 2 MPa), the peak molar fraction of OH radicals is more than twice that of H radicals, indicating that the laminar flame speed is more dependent on OH radicals at higher temperature and pressure. The effect of cyclohexane addition on the surrogate-fuel laminar flame speed and for OH radicals and H radical molar fraction was smaller for both operating conditions.

### 3.3. Effect of Cyclohexane on the Combustion Characteristics of HCCI

In addition to ignition and flame propagation characteristics, fuel combustion in the cylinder is also a major characteristic. In HCCI engines, the mixture reaches the autoignition temperature almost simultaneously in the cylinder while exothermic reactions occur, which can be carried out in a thinner mixture, significantly reducing the generation of NOX and carbon smoke. To investigate the effect of cyclohexane addition on the in-cylinder combustion of surrogate fuels, the phase combustion of four- and five-component surrogate fuels in HCCI engines under lean conditions was investigated. The effect of cyclohexane on fuel autoignition was studied and explained from the perspective of fuel chemistry kinetics. In addition, the effects of engine speed, fuel/air ratio, and intake air temperature are analyzed.

Sjöberg [46] investigated the intake air temperature required for the combustion timing of gasoline (RON = 90.8, MON = 83.4) in a Cummins B-series engine with 102 mm bore, 120 mm stroke, and 192 mm connecting rod length, with fuel ignition by compression. The HCCI model with a zero-dimensional single-zone homogeneous compression ignition was used for the calculations, starting at −180 °CA. Four-component fuel (fuel 1) and five-component fuel (fuel 5) from Table 7 were chosen as surrogate fuels. The combustion phase was defined as the moment when the CO molar fraction reached its peak. The three engine speeds were 600 rpm, 1200 rpm, and 1800 rpm, respectively, and the compression ratio increased with increasing speed, due to the consideration of heat loss during the calculation [27]. It was performed with natural intake and an intake pressure of 0.1 MPa.

**Figure 17:**
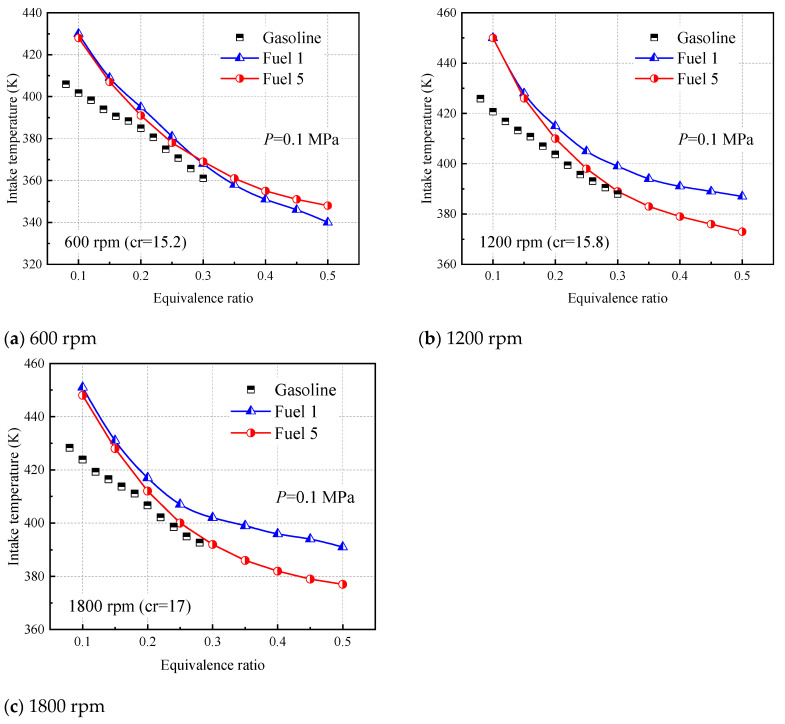
Required intake temperatures at an intake pressure of 0.1MPa to phase combustion at TDC in an HCCI engine.

Figure 17 shows the inlet gas temperatures required for fuel 1 and fuel 5 to burn at positive ignition times in HCCI with equivalent ratios ranging from 0.1 to 0.5, with gasoline experimental data from Sjöberg [46]. The results of the model calculations are recorded in Table 8.

As can be seen from Figure 17, the predictions for both fuel 1 and fuel 5 are higher than the experimental values for very lean conditions with equivalence ratios less than 0.2. This indicates that the model selected in this paper is applicable in the region where the lean limit is above the equivalent ratio of 0.2, which is obviously sufficient for the gasoline engine developed so far. In Figure 17a, there is little difference between the inlet temperatures required for fuel 1 and fuel 5 to reach ignition at the top stop of compression (TDC) at 600 rpm, and fuel 5 predicts higher inlet temperatures than fuel 1 after the equivalence ratio is greater than 0.3. However, at higher speeds of 1200 rpm and 1800 rpm (Figure 17b,c), fuel 5, a five-component surrogate fuel containing cyclohexane, shows a better predictive trend, while fuel 1 requires a higher intake temperature to achieve positive ignition at higher engine speeds. This can be explained in the sensitivity analysis of the two fuels in Figure 6. At lower temperatures, the macromolecular reactions of the fuel dominate, while cyclohexane molecules are more likely to shed H atoms in the low-temperature region, and H radicals collide with O_2_ molecules to produce a large number of OH radicals, making fuel 5 the first to ignite at the same intake temperature. In other words, fuel 5 requires a lower intake temperature than fuel 1 for ignition timing. In addition, the easier ignition of fuel 1 at low rpm (600 rpm) is attributed to the accelerated conversion of fuel molecules during the low-temperature stall phase (cold-flame phase) of the four-component fuel, which releases heat and leads to system temperature rise, as will be further discussed in the later analysis.

**Figure 18:**
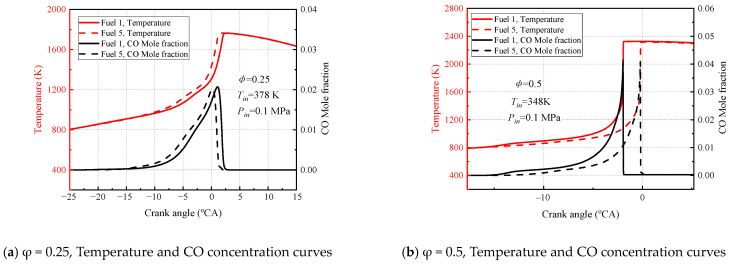

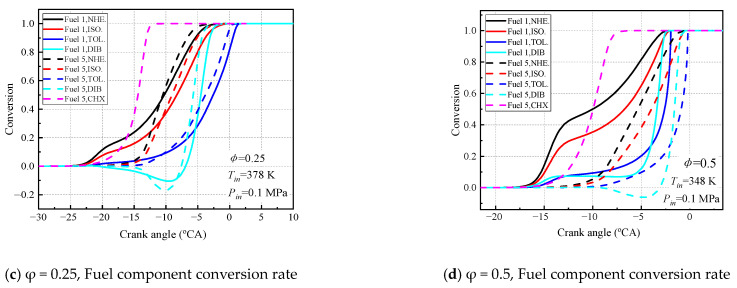
Calculated temperature, CO mole fraction, and each component conversion for fuel 1 and fuel 5 at 600 rpm engine speed.

**Figure 19:**
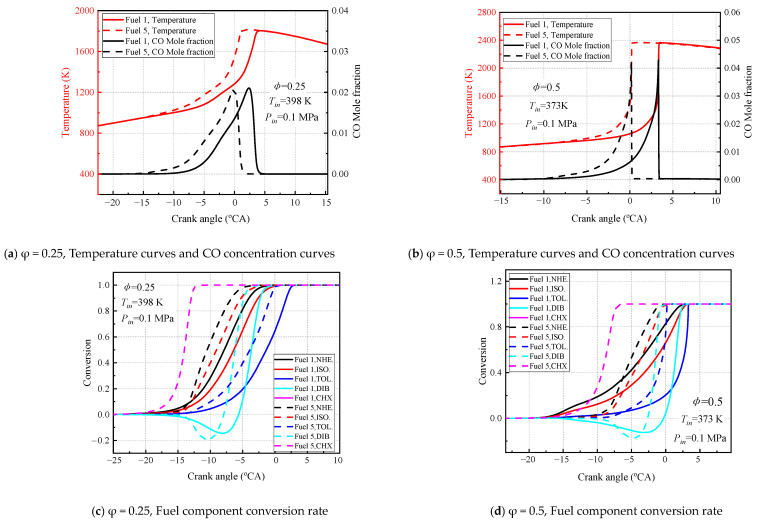
Calculated temperature, CO mole fraction, and each component conversion for fuel 1 and fuel 5 at 1200 rpm engine speed.

Figure 18 and Figure 19 show the variation in temperature, CO molar fraction, and conversion of various surrogate-fuel components calculated for fuel 1 and fuel 5 surrogate fuels at two different engine speeds. Considering the maximum observation of the differences exhibited by the two fuels, two equivalent ratios (0.25, 0.5) were chosen for the calculations, and the intake air temperature was chosen to correspond to the intake air temperature at the fuel 5 ignition timing. 

In Figure 18, the temperatures of fuel 1 and fuel 5 increase with an increasing crankshaft rotation angle at an engine speed of 600 rpm, reaching a maximum at the compression upper stop, followed by a slow decrease in temperature as the mixture expands and does work by combustion, and the peak CO molar fraction also occurs near the compression upper stop and then decreases rapidly (Figure 18a,b). The fuel conversion rate shows that cyclohexane is the fastest, n-heptane is faster than isooctane, and toluene is the smallest (Figure 18c,d). At smaller equivalent ratios (φ = 0.25) the peak CO molar fraction and the moment of maximum temperature gradient for fuel 1 and fuel 5 are not very different (2 °CA), and the inlet temperature of the fuel 5 ignition timing combustion makes the fuel 1 delayed ignition. The reason for this can be found in Figure 18c, although fuel 1 has cold-flame combustion in the crankshaft rotation angle range of −22.5~20 °CA, the conversion of n-heptane and isooctane fuel molecules is not driven at this small equivalent ratio, and the rapid conversion of fuel 5 fuel molecules leads to a delayed ignition of fuel 1 relative to fuel 5. Negative conversion of DIB fuel is also observed in Figure 18c in the −20–8 °CA cranking angle range, which is attributed to the crossover reaction R38. AC_8_H_17_ + O_2_ = JC_8_H_16_ + HO_2_ generated by the isooctane submechanism and the diisobutene submechanism. The conversion of isooctane in the cold-flame stage results in a net production of DIB. The negative conversion of DIB is more pronounced for fuel 5 because the conversion of isooctane in fuel 5 increases rapidly after −15 °CA. When the volume ratio increases to 0.5, a shift in the ignition sequence of fuel 1 and fuel 5 can be observed, and the inlet air temperature of fuel 5 burning at the right time of ignition causes fuel 5 fuel to ignite earlier. The n-heptane and isooctane conversion rates of fuel 1 in Figure 18d explain this change. After the equivalent ratio increases to 0.5, fuel 1 has a large cold-flame combustion phase, which leads to the conversion of a large amount of n-heptane and isooctane, and the OH and H radicals generated drive the system temperature up and, eventually, fuel 1 fuel ignites early. In addition, it can be clearly found that the negative conversion of DIB is suppressed at an equivalence ratio of 0.5, which is due to the sufficient oxidant to promote the conversion of DIB.

In Figure 19, the temperature curves and molar fraction curves for fuel 1 and fuel 5 at 1200 rpm engine speed follow similar trends to the 600 rpm engine speed, with the same moments of peak temperature and peak CO molar fraction, and the fuel-conversion rates show that cyclohexane converts the fastest, n-heptane converts faster than isooctane, and toluene converts the least. Comparing the calculated results for the two surrogate fuels at different equivalence ratios, there are significant differences in the fuel ignition timing and conversion curves. The conversion curves of fuel components showed that the conversion rate of each component in fuel 5 fuel was higher than that of fuel 1 until the compression stop, and the addition of cyclohexane reduced the amount of TRF in the surrogate fuel, especially toluene, which had the lowest conversion rate, making fuel 5 to burn at the right time of ignition. No cold flame combustion region was found for fuel 5 in all operating conditions, as also observed in Figure 18, due to the increased reactivity of the fuel by the addition of cyclohexane. These phenomena indicate that the addition of the cyclohexane component has a great influence on the combustion characteristics of the surrogate fuel in HCCI.

In addition to the effect of cyclohexane, the effects of engine speed, equivalence ratio, and intake temperature were analyzed by comparing Figure 18 and Figure 19. For different engine speeds, it is clearly observed that higher intake air temperature is required for the fuel mixture to achieve ignition timing combustion at higher engine speeds. In addition, increasing engine speed inhibited the cold flame behavior of the fuel 1 four-component surrogate fuel because the fuel residence time was reduced at higher speeds and the response in the low-temperature region could not be maintained. For the equivalence ratio, increasing the equivalence ratio is more favorable for the ignition timing combustion of the fuel, which can be compared with Figure 17. The required intake-gas temperature for the ignition timing of the fuel decreases with increasing the equivalence ratio, and the increase of the oxidizer can promote the oxidation of the fuel in the lean condition. For the effect of inlet gas temperature, it is mainly reflected in the ignition timing of fuel 1 and fuel 5; the higher the inlet gas temperature, the easier the fuel ignites.

**Figure 20:**
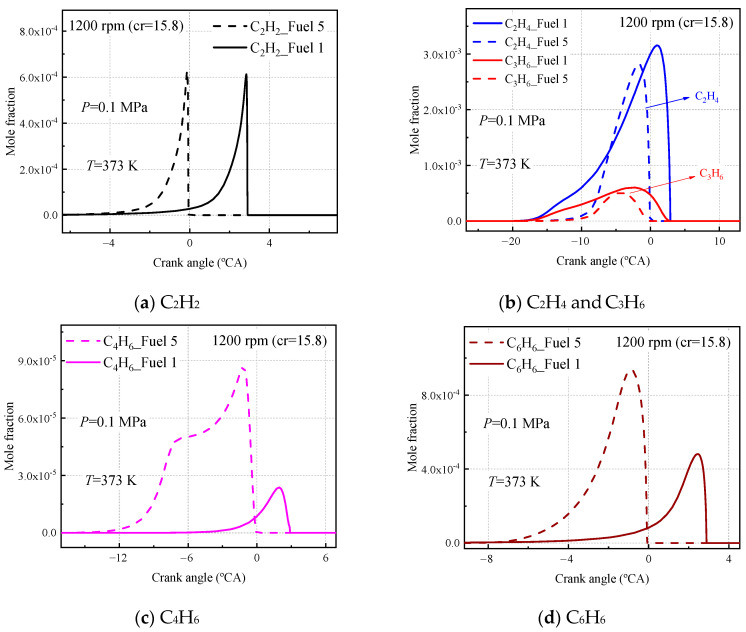
Partial intermediate product mole fractions calculated for fuel 1 and fuel 5 at 1200 rpm.

Finally, to investigate the emissions of both fuel 1 and fuel 5 surrogate fuels in the HCCI engine, the molar fraction production of some important carbon-soot precursors was analyzed at an inlet temperature of 373 K, an inlet pressure of 0.1 MPa, an equivalent ratio of 0.5, and an engine speed of 1200 rpm, as shown in Figure 20. The production of acetylene (C_2_H_2_), ethylene (C_2_H_4_), propylene (C_3_H_6_), 1, 3-butadiene (C_4_H_6_), and benzene (C_6_H_6_) were included.

As seen in Figure 20, the fuel 1 and fuel 5 calculations for ethylene, acetylene, and propylene are the same, and the difference in the corresponding peaks is due to the different moments of ignition for the two fuels at 373 inlet temperatures. In Figure 20b, the C_2_H_4_ and C_3_H_6_ curves calculated for the two fuels differ, with the molar fraction curves for ethylene and propylene calculated by fuel 5 being more concentrated in front of the compression upper stop, while the curves calculated by fuel 1 are more dispersed, due to the cold-flame behavior of fuel 1. The molar fraction curves for C_4_H_6_ and C_6_H_6_ calculated by fuel 5 are clearly different from those of fuel 1 (Figure 20c,d), the 1,3 butadiene molar fraction experienced a rapid increase and then leveled off before growing to a peak, which was not observed in fuel 1. In addition, the peak molar fraction of C_6_H_6_ calculated in fuel 5 was twice as high as that calculated in fuel 1.

**Figure 21:**
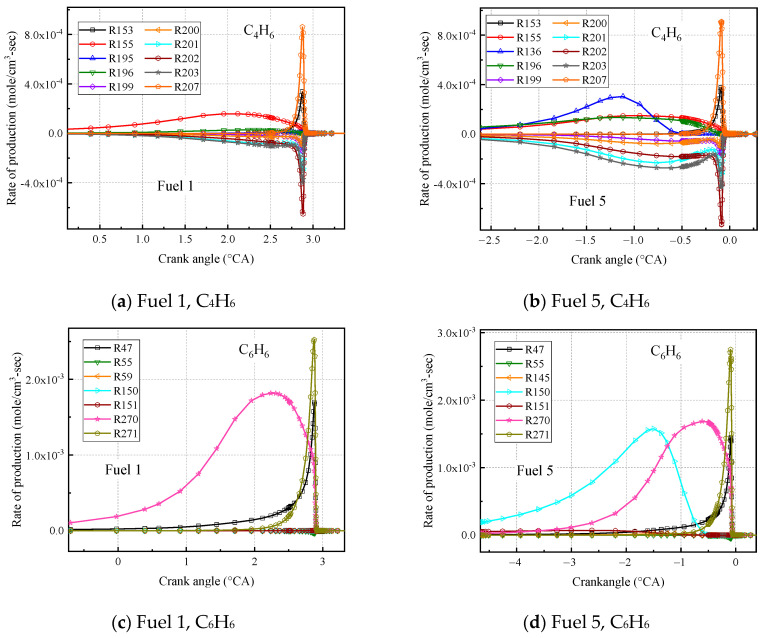
Calculated C_4_H_6_ and C_6_H_6_ reaction rates for fuel 1 and fuel 5 at 1200 rpm.

The analysis of the rate of production (ROP) was further analyzed for C_4_H_6_ and C_6_H_6_ at this working condition, as shown in Figure 21.

Figure 21a,b shows the first ten reactions with faster chemical reaction rates for C_4_H_6_ calculated for the surrogate fuels fuel 1 and fuel 5, where a positive reaction rate indicates fuel production and vice versa for consumption. It can be seen that among the first ten radical reactions calculated for the latter, R136; cC_6_H_10_ = C_4_H_6_ + C_2_H_4_ is the added reaction, which produces a 1, 3 butadiene molecule and an ethylene molecule from the decomposition reaction of cyclohexene C-C bond breakage. In addition, the reaction rate of the radical reaction R196. C_4_H_7_ + O_2_ = C_4_H_6_ + HO_2_ was increased in the fuel 5 calculations, and the R136 and R196 reactions together contributed to the higher molar fraction of C_4_H_6_ calculated for fuel 5. Figure 21c,d shows the first ten reactions with faster chemical reaction rates for the surrogate fuels fuel 1 and fuel 5 calculated for C_6_H_6_. It is observed that the addition of cyclohexane greatly increases the chemical reaction rate for the radical reaction R150. SAXcC_6_H_7_ = C_6_H_6_ + H. The removal of an H atom from SAXcC_6_H_7_ to form C_6_H_6_ is what leads to the increase in the molar fraction of C_6_H_6_ in fuel 5.

From the above analysis, it is evident that the addition of cyclohexane has different effects on the surrogate-fuel ignition, flame propagation, and combustion characteristics in the HCCI cylinder. In the low-temperature reaction stage, the ignition-delay time of the fuel decreased gradually as the proportion of cyclohexane in the surrogate fuel increased, which was caused by the early oxidation and decomposition of cyclohexane molecules to produce more OH-active molecules. In the medium-temperature reaction stage, the addition of cyclohexane suppressed the negative temperature behavior of the surrogate fuels and became more pronounced with increasing pressure because the fuels containing cyclohexane were still dominated by macromolecular reactions in the medium-temperature stage and their ignition delay was less dependent on pressure, which affected the NTC behavior of the surrogate fuels in this region. The role of cyclohexane becomes less pronounced as the combustion characteristics in the high-temperature reaction phase are controlled by the small molecule reactions.

The coupling mechanism considers H_2_/CO/C_1_ as the core mechanism, which responds to the laminar flame speed of the fuel so that the laminar flame speeds of surrogate fuels with different cyclohexane ratios are similar. The small difference is attributed to the fact that the proportion of n-heptane, which has a larger flame speed, is reduced with the addition of cyclohexane.

HCCI engine tests showed that the five-component surrogate fuel containing cyclohexane required lower intake-gas temperature to achieve ignition timing at higher engine speed, and was closer to the in-cylinder ignition of real gasoline. In addition, the addition of cyclohexane results in higher concentrations of 1, 3-butadiene and benzene at ignition timing than in the four-component fuel.

## 4. Materials and Methods

We have described all the materials and methods we used in our previous work. If you want to know more, you can read this literature [30].

## 5. Conclusions

In this study, the effect of cyclohexane addition on the ignition and combustion performance of surrogate fuels are analyzed based on three aspects: ignition delay, laminar flame speed, and in-cylinder combustion characteristics of HCCI engines. The main findings are as follows.

(1) Based on extensive experimental data of real gasoline, our previously constructed simplified kinetic model of CDTRF five-component gasoline surrogate fuel shows good predictive performance in three aspects of fuel ignition delay, laminar flame speed, and in-cylinder combustion characteristics of HCCI engines.
(2) As the proportion of cyclohexane in the surrogate fuel increases, the ignition-delay time of the fuel gradually decreases at low and high temperatures. While at the medium-temperature reaction stage, the isomerization and decomposition reactions of cyclohexane oxide cC_6_H_12_O_2_ dominate the temperature sensitivity of the ignition delay due to the addition of cyclohexane, which affects the small molecule reactions that promote the formation of reactive radicals such as OH molecular reactions, thus inhibiting the NTC behavior of surrogate fuels. This trend becomes more and more obvious with the increase in pressure.
(3) The laminar flame speed of the surrogate fuels increases with the increase of the cyclohexane ratio. This is due to the fact that the laminar flame speed of cyclohexane is higher than that of chain and aromatic hydrocarbons, and the addition of cyclohexane dilutes the proportion of chain and aromatic hydrocarbons in the mixture. From a microscopic point of view, the concentration of reactive radicals such as H and OH generated by each component varies. The C-C and C-H in toluene are more stable than other hydrocarbons, so cyclohexane addition increases the laminar flame speed of the fuel mixture.
(4) Engine simulation studies show that at relatively high engine speeds, the five-component surrogate fuels containing cyclohexane require lower intake-gas temperatures to achieve ignition timing and are closer to the in-cylinder ignition of real gasoline; the addition of cyclohexane results in higher concentrations of 1,3-butadiene and benzene at ignition timing than the four-component fuels.

In this paper, some discussions on the effects produced by cyclohexane in gasoline components are made at the kinetic mechanism level. In future research, the five-component gasoline kinetic model constructed by our group will be considered for the three-dimensional numerical simulation of engines. The effect of the cyclohexane component in the five-component gasoline surrogate fuel on engine combustion and emissions will be studied in a wider range and compared with existing studies.

## Figures and Tables

**Table 1:** Physical and chemical properties of components of gasoline surrogate fuels.

Component	Molecular Formula	RON	MON	Calorific Value (kJ/kg)	H/C	Density (kg/m^3^)	Molecular Weight
N-Heptane	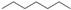	0	0	44.566	2.28	684	100.2
Isooctane	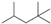	100	100	44.427	2.25	692	114.23
Toluene	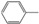	120	103.5	40.589	1.14	867	92.14
DIB	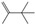	113.75	107.2	43.72	2	715	112.21
CHX	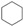	83	77.2	43.08	2	773	84.16

**Table 2:** Three surrogate fuels of FACE G gasoline (by volume fraction).

Mixture	Isooctane (%)	N-Heptane (%)	Toluene (%)	DIB (%)	CHX (%)	RON	Ref.
TRF-G	58.1	11.6	30.3	0	0	96.8	[28]
DTRF-G	25	20	45	10	0	94.6	[31]
CDTRF-G	23.75	19	42.75	9.5	5	94	[30]

**Table 3:** Three surrogate fuels of Exxon 708629-60 gasoline (by volume fraction).

Mixture	Isooctane (%)	N-Heptane (%)	Toluene (%)	DIB (%)	CHX (%)	RON	Ref.
TRF-Exxon	33.33	33.33	33.33	0	0	78	[34]
DTRF-Exxon	25	20	45	10	0	94.6	[31]
CDTRF-Exxon	65.31	8.11	14.52	5.36	6.7	95	[30]

**Table 4:** The size of the Volvo TD100 engine used in the experiment.

Parameters	Bore (mm)	Stroke (mm)	Displacement (cm^3^)	Connecting Rod Length (mm)
Volvo TD100	120.6	140.0	1600	260.0

**Table 5:** Engine operating conditions.

Working Condition	C	Equivalence Ratio	*P*_in_/MPa	*T*_in_/K	Speed/rpm
1	22.4	0.333	0.101	303	1000
2	20	0.333	0.101	343	1000
3	17.7	0.333	0.101	383	1000

**Table 6:** Three surrogate fuels of Grön 98 MK1 gasoline (by volume fraction).

Mixture	Isooctane (%)	N-Heptane (%)	Toluene (%)	DIB (%)	CHX (%)	RON	MON
TRF-Grön	11.9	20.5	67.6	0	0	98.5	87.6
DTRF-Grön	10.6	19.8	61.8	7.8	0	98.5	87.9
CDTRF-Grön	5.3	18	65.6	1.6	9.5	98.5	87.6

**Table 7:** Gasoline fuel surrogates with different cyclohexane ratios (by volume fraction).

Mixture	Isooctane (%)	N-Heptane (%)	Toluene (%)	DIB (%)	CHX (%)	RON
Fuel 1	25	20	45	10	0	94.6
Fuel 2	23.75	19	42.75	9.5	5	93.9
Fuel 3	22.5	18	40.5	9	10	93.2
Fuel 4	21.25	17	38.25	8.5	15	92.6
Fuel 5	20	16	36	8	20	92.0

**Table 8:** Required intake temperatures at an intake pressure of 0.1 MPa to phase combustion at TDC in an HCCI engine (K).

Equivalence Ratio	Fuel 1			Fuel 5		
	600 rpm	1200 rpm	1800 rpm	600 rpm	1200 rpm	1800 rpm
0.1	430	450	451	428	450	448
0.15	409	428	431	407	426	428
0.2	395	415	417	391	410	412
0.25	381	405	407	378	398	400
0.3	368	399	402	369	389	392
0.35	358	394	399	361	383	386
0.4	351	391	396	355	379	382
0.45	346	389	394	351	376	379
0.5	340	387	391	348	373	377

## Data Availability

Data are contained within the article.

## Sample Availability

Samples are not available from the authors.
